# Serum retinol binding protein as a novel marker for clearance and dosage optimization: pharmacokinetics study of voriconazole in a cirrhosis population

**DOI:** 10.3389/fphar.2025.1543323

**Published:** 2025-05-21

**Authors:** Juping Yun, Haihong Bai, Zihe Wang, Yingmin Ma, Wei Liu

**Affiliations:** ^1^ Department of Pharmacy, Beijing YouAn Hospital affiliated to Capital Medical University, Beijing, China; ^2^ Department of Respiratory and Critical Care Medicine, Beijing YouAn Hospital affiliated to Capital Medical University, Beijing, China

**Keywords:** voriconazole, retinol binding protein, population pharmacokinetics, cirrhosis, therapeutic drug monitoring, dosage recommendation

## Abstract

**Background and Objective:**

Voriconazole (VRC) exhibits nonlinear pharmacokinetic (PK) characteristics and a narrow therapeutic window. Consequently, standardized dosage regimens are insufficient to achieve the targeted therapeutic exposure in patients with cirrhosis. While numerous population pharmacokinetic (PPK) studies on VRC have been conducted, data on the cirrhosis demographic remain limited.This study aimed to explore the PK characteristics of VRC and its covariates in a cirrhosis population, with the objective of recommending individualized dosing regimens.

**Methods:**

Data collected from routine therapeutic drug monitoring (TDM) of patients with recorded VRC plasma concentrations during a period of therapy between September 2022 and August 2024 were included. A population pharmacokinetic (PPK) model was constructed using nonlinear mixed-effects modeling (NONMEM). Monte Carlo simulation was used to predict the target trough concentrations of VRC under steady-state conditions based on the final model parameters, thereby facilitating tailored dosage recommendations.

**Results:**

A total of 151 trough concentrations were obtained from 78 patients enrolled in the PPK study of VRC. A one-compartment model featuring first-order absorption and first-order elimination was optimal in describing the PK characteristics, additionally incorporating Child-Pugh grades and retinol-binding protein (RBP) as covariates affecting the central ventricular clearance rate (CL) of VRC. In the final model, the CL was determined as 6.96 L/h. For patients classified as Child-Pugh A and B with RBP ≥25 mg/L, the recommended dosages were 400 mg/d and 200 mg/d, respectively. At RBP levels <25 mg/L, the recommended dosages for Child-Pugh A and C patients were 200 mg/d and 100 mg/d, respectively, while for Child-Pugh B patients, both 200 mg/d and 100 mg/d were recommended.

**Conclusion:**

Our results support the utility of RBP as a novel marker associated with VRC clearance. This biomarker may offer a practical option for VRC dosage optimization. The clinical dosage of VRC could be tailored according to the Child-Pugh grades and RBP levels of patients. While numerous unexplained factors potentially influence the pharmacokinetic properties of VRC, the application of PPK model-guided TDM is crucial for achieving precision in individualized medication regimens.

## 1 Introduction

Liver cirrhosis is currently one of the leading contributors to morbidity and mortality worldwide (Asrani et al., 2019). The condition is marked by cirrhosis-associated immune dysfunction (CAID), alongside gut dysbiosis and impaired intestinal barrier, which collectively create susceptibility to infections (Maraolo et al., 2020). Consequently, patients with liver cirrhosis are particularly prone to developing infectious diseases. In particular, fungal infections in cirrhosis are associated with significant mortality and poor outcomes, with *Candida spp*. and *Aspergillus spp*. identified as the predominant pathogens (Fernández et al., 2021; Gustot et al., 2014; Verma et al., 2021).

Voriconazole (VRC), a first-line therapeutic agent for invasive aspergillus (IA) treatment, is primarily metabolized by the liver, and its dosage in patients requires adjustment based on liver function. According to the package insert for VRC, individuals with mild to moderate cirrhosis (Child-Pugh Class A and B) should be administered the same loading dose as those with normal hepatic function, but half the maintenance dose. However, no specific dosage guidelines have been provided for individuals diagnosed with Child-Pugh C cirrhosis. Population pharmacokinetic (PPK) analysis is utilized to evaluate pharmacokinetic (PK) characteristics and identify the measurable factors contributing to patient-related and clinical-related PK variabilities. Monte Carlo simulation is a valuable tool for determining dosing regimens (Tang et al., 2019; Trang et al., 2017). VRC exhibits nonlinear PK characteristics and possesses a narrow therapeutic window. Therefore, standardized dosage regimens are insufficient to achieve the targeted therapeutic concentrations in different clinical settings, particularly in patients with cirrhosis. While several PPK studies have provided dosage recommendations for VRC (Wang et al., 2018a; Wang et al., 2018b; Wang et al., 2021), data specifically pertaining to the cirrhosis population are limited at present (Ren et al., 2019).

Our hospital is a specialized medical institution dedicated to the treatment of liver diseases, liver transplantation, and AIDS-related infectious diseases. A significant proportion of the patients present with a combination of cirrhosis and immune dysfunction, and therefore, determination of the accurate dosage regimen for VRC therapy is crucial for reducing the morbidity and mortality associated with IA, especially for patients classified as Child-Pugh C. This study aims to: 1) establish a PPK model for VRC in patients with cirrhosis based on real-world clinical data, 2) identify factors significantly associated with the PK parameters of VRC, and 3) develop optimized dosage regimens for patients with different degrees of liver cirrhosis based on the final PPK model.

## 2 Materials and methods

### 2.1 Patients

This single-center retrospective and observational study was conducted at Beijing You’An Hospital affiliated to Capital Medical University. Patients with recorded VRC plasma concentrations during therapy between September 2022 and August 2024 were enrolled. Concentration data categorized as below the lower limit of quantification (BLQ) were excluded.

### 2.2 Blood sampling and analytical assays

The steady trough concentrations (C_min,ss_) were collected according to the individualized medication guidelines for VRC (Chen et al., 2018). Ideally, the initial blood sample was obtained no earlier than immediately before the fifth dose when the loading dose of VRC was administered in the first day. If this timing was not feasible, the initial blood sample was collected no earlier than immediately before the 11th dose.

A validated ultra-high performance liquid chromatography tandem mass spectrometry (UPLC-MS/MS) technique was employed to measure VRC concentrations according to the guideline of ICH (2022). Analysis was performed on Waters Acquity UPLC2695 tandem TQD mass spectrometry (MS) system, data acquisition was performed by using Masslynx™ V4.1 software (Waters Corp.). ACQUITY UPLC^®^BEH C_18_ (2.1 mm × 50 mm, 1.7 μm) was utilized for chromatographic separation. The mobile phase consisted of water solution containing 0.1% formic acid (eluent A) and methanol solution containing 0.1% formic acid (eluent B) at a flow rate of 0.4 mL/min. The run time was 2.5 min with the following gradients: 0–0.2 min, B 5%; 0.5–0.8 min, B 5%→50%; 0.8–1.5 min, B 50%→95%; 1.5–1.51 min, B 95%; 1.51–2.50 min, B 95%→5%. Column oven and autosampler were set at 40^o^C and 4^o^C, respectively. Multiple reaction monitoring (MRM) scan type was carried out for the determination of VRC andVRC-d3 (internal standard, IS) using an ESI source in positive-ion mode. The optimal MS conditions were as follows: capillary voltage of 0.5 kV, cone voltage of 30 V, desolvation temperature of 500°C at a desolvation gas flow of 1000 L/h, and a cone gas flow of 20 L/h. The optimal collision energy and cone energy of VRC were 30 V and 34V; and of IS were 30V and 16 V. The optimized m/z values were 350.10→127.03 and 353.10→284.05 for VRC and IS, respectively.

The linearity range was 0.1–15.0 mg/L (correlation coefficient R^2^ > 0.99). The lower limit of quantification was 0.1 mg/L. The assay precision (intra-day and inter-day variability) was 3.2%–5.3%. The intra-day and interday accuracies were within 100% ± 15%, whereas the intra-day and inter-day precisions -were less than ±15%.

### 2.3 Data collection and analysis

The datasets for study were derived from the laboratory information system and medical records. The information included classification covariates (Sex, Proton pump inhibitors (PPI) combination, Tuberculosis drugs combination, After liver transplantation, and Child-Pugh Classification) and continuous covariates (Age, Height, Weight (WT), Body mass index (BMI), Body surface area (BSA), C-reactive protein (CRP), White blood cell count (WBC), percentage of neutrophils (NE%), hemoglobin (HGB), procalcitonin (PCT), albumin (ALB), alanine aminotransferase (ALT), aspartate aminotransferase (AST), total bilirubin (TBIL), direct bilirubin (DBIL), alkaline phosphatase (ALP), total bile acid (TBA), creatinine (CR), creatinine clearance (CRCL), retinol-binding protein (RBP), estimatedglomerular filtration rate (eGFR), and γ-glutamyl transpeptidase (GGT)). Additionally, data on VRC dosing, delivery route, start and end times, and time of sampling were obtained. The Child-Pugh score was calculated based on the severity of hepatic encephalopathy, amount of ascites, serum bilirubin, serum albumin, and prothrombin time.

For continuous variables, the mean (standard deviation,SD) and median (Min, Max) values were calculated, while for categorical variables, the number of cases (expressed as a percentage, %) was determined.

### 2.4 Data analysis software

The VRC PPK model was constructed using nonlinear mixed-effects modeling software, specifically, NONMEM (version 7.5.0; ICON Development Solutions, Ellicott City, MD, United States), gFortran (version 4.6.3, https://gcc.gnu.org/fortran), and Perlspeaks-NONMEM (version 5.2.6; https://uupharmacometrics.github.io/PsN).

R software (version 4.2.2; https://www.r-project.org) was adopted to organize the raw data, generate a group PK data file, conduct exploratory data analysis, and evaluate and visualize the model.

### 2.5 PPK model

The basic model includes structural and random-effects components. The structural model encompasses the number of compartments, as well as absorption and elimination modes. The random-effects model generally includes inter-individual variability (IIV) and residual unexplained variability (RUV), and may also introduce inter-scenario variability (IOV) where necessary. Both IIV and IOV are represented by an exponential model. The residuals are structured to be additive, proportional, and mixed.

Forward inclusion and backward exclusion were used to screen covariate and parameter combinations for inclusion in the covariate model. The statistical criteria for incorporation of a covariate in the model were established as decrease >6.63 in the objective function value (OFV) (p < 0.01, chi-squared distribution with one degree of freedom) in the forward step and increase >10.83 in the OFV (p < 0.001, chi-squared distribution with one degree of freedom) in the backward step.

For continuous covariates, the following formula was used:

$$P_{i}=P_{TV}\times {\left(\frac{COV}{{COV}_{med}}\right)}^{\theta }$$

For categorical covariates, taking gender as an example, the following formula was applied:

$$\left\{\begin{array}{l} P_{i}\left(\text{male}\right)=P_{TV}\\ P_{i}\left(\text{female}\right)=P_{TV}\times \theta \end{array}\right.$$

***P***_***i***_ represents parameters for the *i*th individual, ***P***_***TV***_ represents the typical values of the parameters, ***COV*** is the continuous covariate, ***COV***_***med***_ refers to median value of continuous covariates, and ***θ*** is the coefficient quantifying the influence ofthe covariate on parameters.

The basic model examined one-compartment with linear elimination, two-compartment with linear elimination, one-compartment with linear elimination plus bioavailability, and one-compartment with linear elimination plus absorption delay (D1).

### 2.6 Model evaluation

The predictive performance of the final model was evaluated using the goodness-of-fit (GOF) plot, bootstrap method, prediction-corrected visual predictive checks (pcVPCs) and external verification.

GOF scatter plots, which include the population predictionversus dependent variable (PRED vs. DV) scatter plot, individual prediction versus dependent variable (IPRED vs. DV) scatter plot, conditional weighted residual versus population predicted value (CWRES vs. PRED) scatter plot, along with histograms and Quantile-Quantile plots, including CWRES and CIWRES, were adopted to assess the fitness of the final model. To assess the accuracy and stability of the final model, the bootstrap method was introduced with 1,000 repetitions. In addition, prediction-corrected visual predictive checks (pcVPCs) were simulated 1000 times to graphically assess the predicted performance of the final model.

Additionally, external validation was conducted using data collected from 22 subjects who met the inclusion criteria of our study. The precision and accuracy of the predictive ability of the model were evaluated using the prediction error (PE) and absolute prediction error (APE):

$${PE}_{j}=\frac{{pred}_{j}-{obs}_{j}}{{obs}_{j}}\times 100\%$$

$$\left.{APE}_{j}\left.=\right|{PE}_{j}\right|$$

${\text{pred}}_{j}$ and ${\text{obs}}_{j}$ represent the ***j*th** predicted value and observed value, respectively.

To assess the performance of model, the percentage of PE that falls within the intervals of ±20% (F_20_) and ±30% (F_30_) was additionally used.

### 2.7 Dosing regimen simulation

Monte Carlo simulation was applied to predict the target C_min,ss_ of VRC using the final model parameters. In total, 1,000 replicates of C_min,ss_ were simulated for each dosage regimen. The target trough concentration range was established as 0.5–5.0 mg/L (Chen K et al., 2018), with different loading and maintenance doses simulated for each sub-population stratified by the covariates included in the final model. IIV, RUV, and IOV were included in this simulation.

## 3 Results

### 3.1 Patient demographics

The original dataset for the PK analysis consisted of 80 patients, of which 2 patients lacked records of observable concentrations after administration (the concentration lower than 0.1 mg/L). Finally, a total of 151 C_min,ss_ obtained from 78 patients were included in the PK study of VRC, 57 (73.1%) of whom were males. The average age of the cohort was 54.4 ± 14.0 years and average weight was 64.9 ± 14.3 kg. The distribution of patients within the Child-Pugh A, B, and C categories were 30 (38.5%), 20 (25.6%), and 28 (35.9%), respectively.

External validation was conducted using a total of 48 observable records collected from 22 additional subjects, and 45.5% were male. The age was 51.1 ± 9.76 years. The patients of Child-Pugh A, B and C was 11 (50.0%), 6 (27.3%), and 5 (22.79%), respectively. The RBP was 31.4 ± 23.1 mg/L.

Further details of the patient characteristics and covariates are presented in Supplementary Tables S1, S2.

### 3.2 VRCdosing and C_min,ss_

The VRC dosing regimens of our study were collected and shown in Table 1. Of all the **C**_**min,ss**_ which included in the PK model, 56.95% (86/151) was in the target range (0.5–5.0 mg/L), with 3.31% (5/151) was below 0.5 mg/L, and 39.74% (60/151) was above 5.0 mg/L.

**TABLE 1:** The VRC dosing in the PK model.

Child-Pugh classification	The dosage in the first 24 h	Maintain dosage	PK model (N = 78)
A	400 mg, q12 h	200 mg, q12 h	13
	200 mg, q12 h	200 mg, q12 h	15
	150 mg, q12 h	150 mg, q12 h	1
	100 mg, q12 h	100 mg, q12 h	1
B	400 mg, q12 h	200 mg, q12 h	12
	200 mg, q12 h	200 mg, q12 h	5
	200 mg, q12 h	100 mg, q12 h	2
	100 mg, q12 h	100 mg, q12 h	1
C	400 mg, q12 h	200 mg, q12 h	20
	200 mg, q12 h	200 mg, q12 h	6
	200 mg, q12 h	100 mg, q12 h	1
	100 mg, q12 h	100 mg, q12 h	1

### 3.3 PPK model development

The concentrations obtained in this study were all C_min,ss_, which posed a challenge in accurately estimating the absorption rate constant (Ka). The result of our sensitivity analysis of Ka showed that the fixed Ka values have no significant impact on CL and V estimation (Supplementary Table S3). Thus, the absorption rate constant was fixed at 1.1 h^−1^ based on an earlier literature report (Shi et al., 2019). Subsequently, the clearance (CL) and volume of distribution (Vc) of VRC were characterized and estimated.

A one-compartment model with linear elimination including Child-Pugh grade on the CL of VRC (ΔOFV = −38.366) was used to describe the PK characteristics of VRC in the basic model. After the forward inclusion and backward exclusion covariate modeling procedure, the Child-Pugh classification and RBP which showed significant influence on the CL of VRC were included in the final model. The process of development of the PPK model for VRC is illustrated in Supplementary Table S4.

The final model equation is as follows:

$${\text{CL}}_{i}\left(L/\text{hr}\right)=\left\{\begin{array}{l} \text{CPugh}=A,6.96\cdot \exp\left(\eta_{\text{CL},i}\right)\cdot {\left(\frac{\text{RBP}}{23.9}\right)}^{0.428}\\ \text{CPugh}=B,6.96\cdot \exp\left(\eta_{\text{CL},i}\right)\cdot {0.673\cdot \left(\frac{\text{RBP}}{23.9}\right)}^{0.428}\\ \text{CPugh}=C,6.96\cdot \exp\left(\eta_{\text{CL},i}\right)\cdot 0.581\cdot {\left(\frac{\text{RBP}}{23.9}\right)}^{0.428} \end{array}\right\}$$

$${\text{VC}}_{i}\left(L\right)=745\cdot \exp\left(\eta_{\text{VC},i}\right)$$

$$\text{Ka}\left(1/\text{hr}\right)=1.1$$

**CL** represents the clearance rate, **VC** the central chamber distribution volume, **Ka** the absorption rate constant, $\eta_{\text{CL},i}$ the inter individual variation of CL, $\eta_{\text{VC},i}$ the inter individual variation of V_C_, and i the individual.

### 3.4 Model evaluation

**FIGURE 1:**
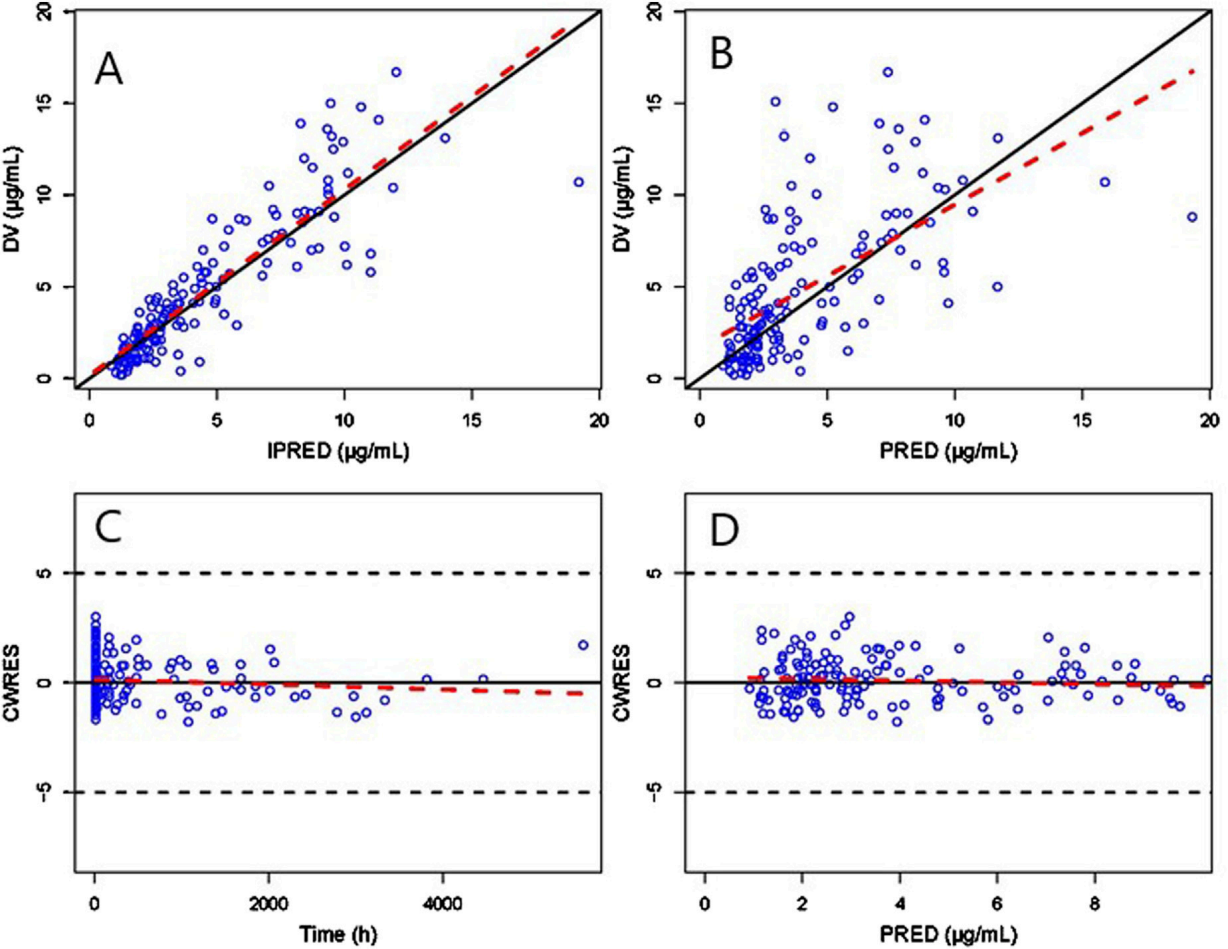
GOF plot of the final Pop-PK model. **(A)** Scatter plot of observed and individual predicted values. **(B)** Scatter diagram of observed and population predicted values. **(C)** Scatter plot of conditional weighted residuals and time after dose. **(D)** Scatter plot of conditional weighted residuals and predicted population values. The solid black lines serve as reference lines, the dashed black lines are auxiliary lines with | CWRES | = 5, and the red dashed lines indicate local weighted linear regression lines.

**FIGURE 2:**
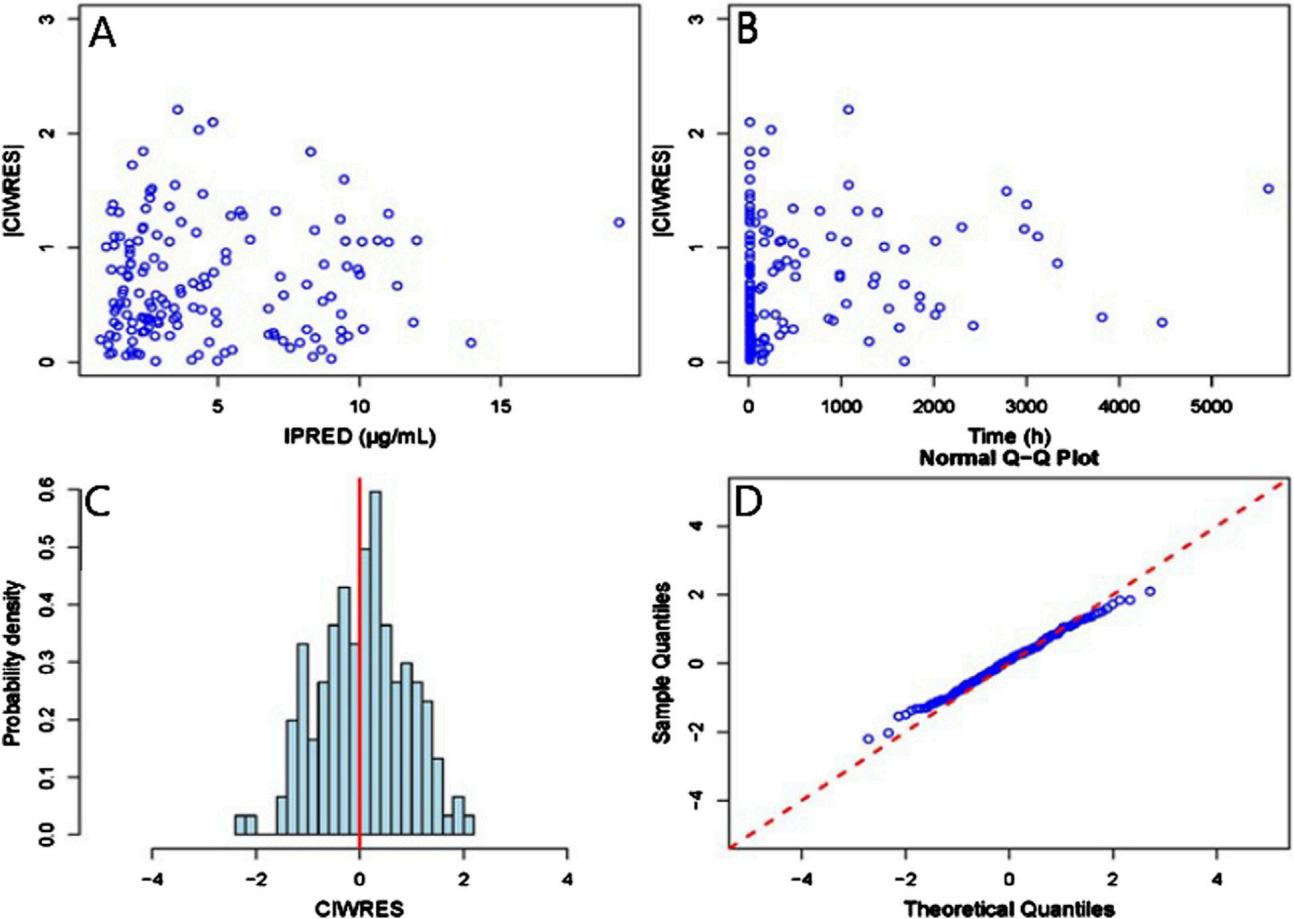
CIWRES diagnostic plot of the final Pop-PK model. **(A)** Scatter plot of absolute values of conditional individual weighted residuals and individual predicted values. **(B)** Scatter plot of absolute values of conditional individual weighted residuals and time in the model. **(C)** Histogram of the absolute values of conditional individual weighted residuals, with the red solid line serving as a reference. **(D)** Q-Q plot of conditional weighted residuals and predicted population values, with the reference line marked by the red dashed line.

**FIGURE 3:**
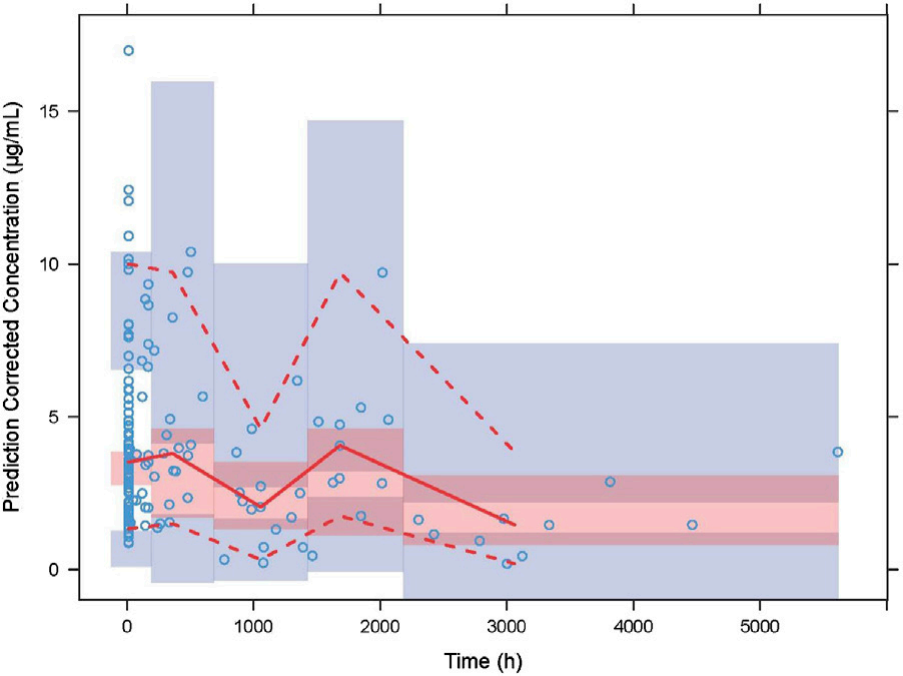
Prediction-corrected visual predictive checks (VPCs) of the final model. The blue hollow dots represent the original observed blood drug concentration of VRC, the solid red line represents the median observed plasma concentration, and the red area represents the 95% confidence interval of the simulation-based median. The red dotted line represents the 5% and 95% percentiles of observed plasma concentration,and the blue area is the 95% confidence interval for the corresponding percentile predicted by the model.

The GOF chart of the final PK model is illustrated in Figure 1. A strong correlation was noted between the observed and predicted individual concentration values, with no significant deviations in the conditional weight residual graph in relation to time and predicted concentration. The diagnostic graph of the conditional individual weighted residuals (CIWRES) of the final model is shown in Figure 2, revealing a basically symmetrical distribution around zeroin close alignment with the theoretical distribution (−2 to 2). The pcVPC visual verification diagram of the final model (Figure 3) shows that the majority of VRC DVs were covered in the 90% prediction interval (PI), indicating that the final model demonstrates satisfactory predictive performance.

The PK parameters of VRC and bootstrap results are presented in Table 2. The median value obtained from bootstrap parameter estimation was similar to that of the final PPK model. The estimated parameters from the final PPK model were within the 95% percentile interval (CI) of bootstrap estimation parameters (represented by the 2.5–97.5^th^ percentile interval) and the 95% CI of the final model parameter estimation was similar to the 95% percentile interval derived from bootstrap estimation, in addition, the bootstrap analysis achieved a success rate of 99.8%, which indicating that the final model was credible, with high predictive accuracy and good stability.

**TABLE 2:** Pharmacokinetic parameters of VRC and bootstrap results.

Parameter	Final model		Bootstrap		
	RSE%	95% CI	Median	2.5th-97.5th CI	RSE%
CL (L/h)	6.96	5.7–8.5	6.95	5.67–8.55	−0.144
Vc (L)	745	532–1040	755	485–1100	1.34
Ka (Fixed, 1/h)	1.1	—	1.1	—	—
Child-Pugh B on CL	0.673	0.514–0.881	0.674	0.491–0.869	0.149
Child-Pugh C on CL	0.581	0.457–0.739	0.575	0.431–0.744	−1.03
RBP on CL	0.428	0.288–0.568	0.426	0.258–0.566	−0.467
IIV of CL	45.9	31.1–58	44.8	27.1–57	−2.4
RSV_CV (%)	36.1	23.4–45.3	34.4	24.2–44.5	−4.71
RSV_SD (mg/L)	0.634	0.00–0.994	0.639	0.236–1.15	0.789

CL: clearance; Vc: distribution volume of the central compartment; RBP: retinol-binding protein; RSV_CV: proportional residual variation; RSV_SD: additive type residual variation; RSE: relative standard error.

The prediction error test results of the PPK model are shown in Table 3. The mean percentage of individual relative errors (MPE%) was within ±15% while the mean absolute percentage of individual relative errors (MAPE%) was within 30%. Furthermore, F_20_ and F_30_ exceeded 30% and 50%, respectively, indicating good predictive performance of the population PK model.

**TABLE 3:** Prediction error test value of the PPK model of VRC.

Statistical terms	PE%	APE%
Mean value (Mean)	−0.84%	28.89%
Median value (Median)	−2.29%	27.98%
F_20_	31.25%	
F_30_	56.25%	

PE: prediction error; APE: absolute prediction error; F20: Percentage of PEs, that fall within ±20%; F30: Percentage of PEs, that fall within ±30%.

### 3.5 Simulations

#### 3.5.1 Evaluation of VRC based on the Child-Pugh classification system

**FIGURE 4:**
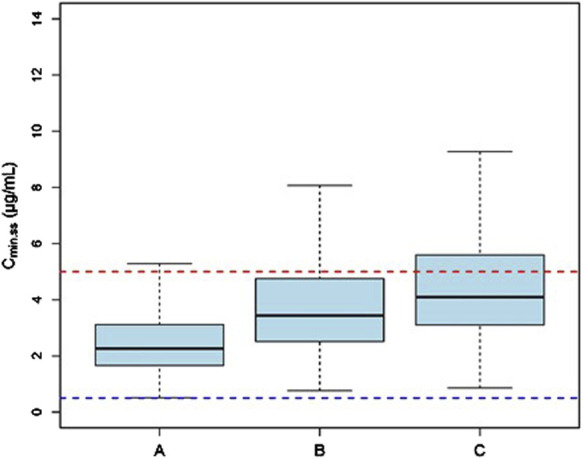
The C_min,ss_ distribution of subjects with different Child-Pugh grades receiving a dosage of “200 mg bid”. **(A)** The C_min,ss_ distribution in subjects with Child-Pugh A. **(B)** The C_min,ss_ distribution in subjects with Child-Pugh B. **(C)** The C_min,ss_ distribution in subjects with Child-Pugh C. The blue and red dotted lines represent the trough concentration range of 0.5 and 5.0 μg/mL, respectively.

Monte Carlo simulations of the VRC steady-state trough concentrations under different dosage regimes were conducted based on the final PK model. The results showed a positive correlation of VRC exposure with Child-Pugh classification of patients under the “200 mg bid” dosage regime (Figure 4). In addition, the distribution and standard-reaching rates of the C_min,ss_ were dependent on the daily dose, despite different administration frequencies (Table 4). A comparative analysis of the compliance rates across different regimes based on Child-Pugh grades was additionally performed. For patients classified as Child-Pugh A, the compliance rates for doses of 400 mg and 200 mg were similar (95.9% vs. 94.8%). However, the rates of C_min,ss_>5.0 mg/L under the 400 mg and 200 mg regimes were 4.1% and 0%–0.1% and C_min,ss_ rates <0.5 mg/L were 0% and 5.2%–5.6%, respectively. For Child-Pugh B and C groups, 200 mg/d was the optimal regime, with compliance rates of 98.1%–99% and 98.2%–98.3%, respectively (Supplementary Table S5).

**TABLE 4:** Comparative analysis of exposure under different dosing regimens categorized by Child-Pugh grades.

Child-Pugh grade	Regime 1	Regime 2	C_min,ss_ (mean [95%CI])
A	200 mg QD	100 mg BID	95.8% (91.8%–100%)
A	100 mg QD	50 mg BID	95.3% (91.4%–99.4%)
B	200 mg QD	100 mg BID	96.3% (92.4%–100.2%)
B	100 mg QD	50 mg BID	95.9% (92%–100%)
C	200 mg QD	100 mg BID	94.1% (90.4%–98%)
C	100 mg QD	50 mg BID	95.6% (92%–99.5%)

C_min,ss_: Target trough concentration under steady-state conditions.

#### 3.5.2 Exposure of VRC in relation to RBP

Based on the final model, RBP was identified as a significant covariate contributing to the clearance of VRC. Since the research data on drug PK using RBP was limited, we conducted ananalysis based on the enrolled patients’ RBP level (Supplementary Table S6). Thus, we conducted simulations with RBP level classified at 25 mg/L based on our clinical RBP distribution analysis result and previous study (Zhang et al., 2014).

**FIGURE 5:**
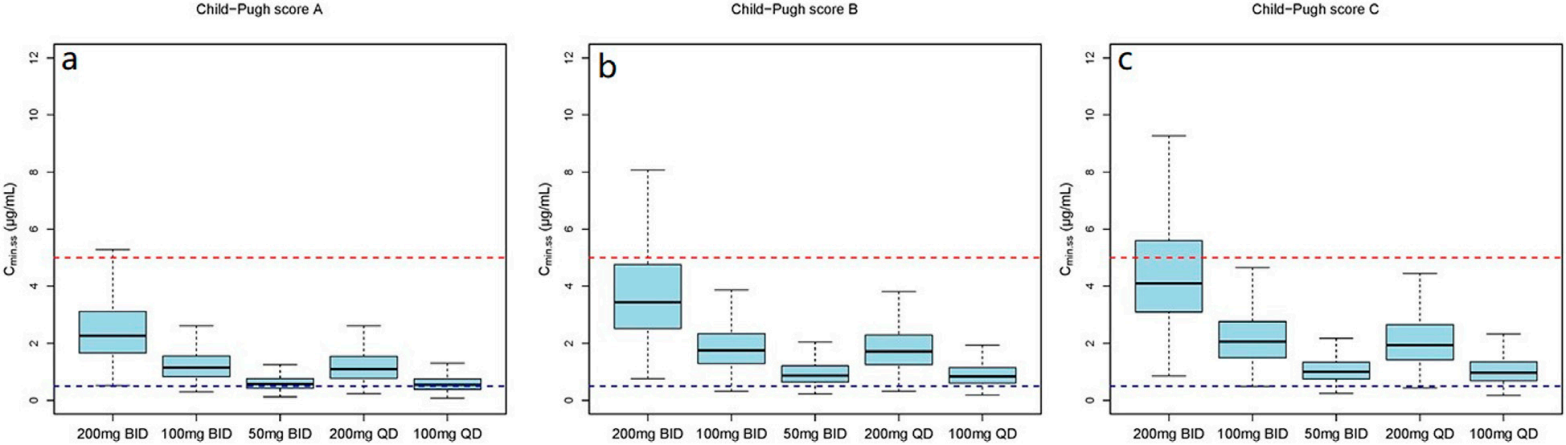
The C_min,ss_ distribution under different dosage regimens. **(a)** The C_min,ss_ distribution in subjects with Child-Pugh score A; **(b)** The C_min,ss_ distribution in subjects with Child-Pugh score B; **(c)** The C_min,ss_ distribution in subjects with Child-Pugh score C.

**FIGURE 6:**
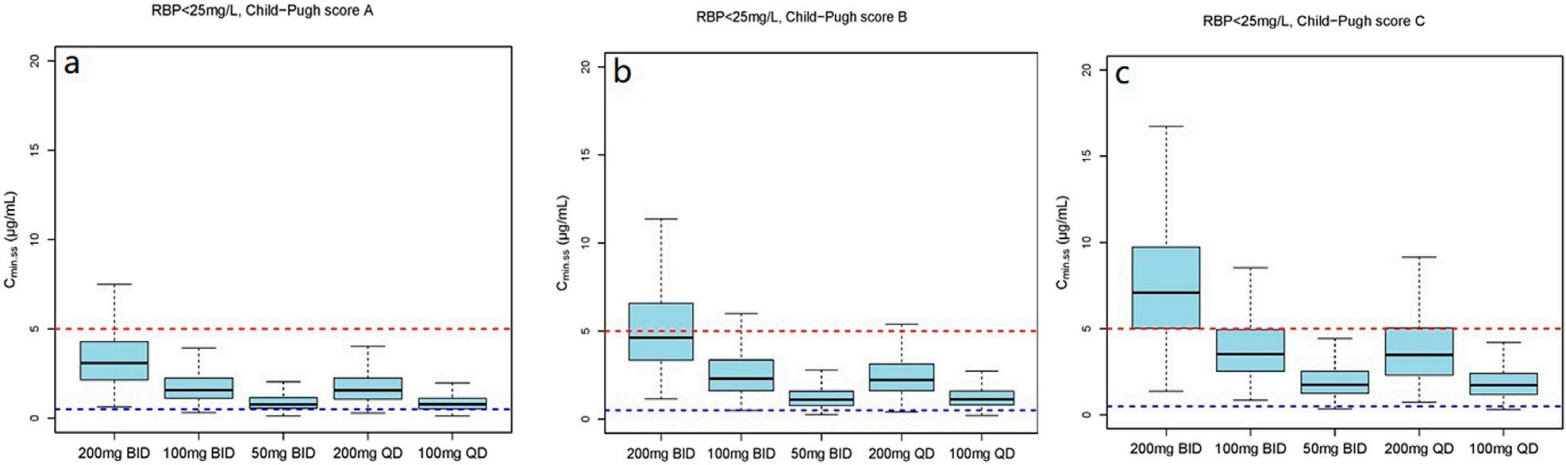
The C_min,ss_ distribution under different dosage regimens in subjects with RBP <25 mg/L. **(a)** The C_min,ss_ distribution in subjects with Child-Pugh score A; **(b)** The C_min,ss_ distribution in subjects with Child-Pugh score B; **(c)** The C_min,ss_ distribution in subjects with Child-Pugh score C.

**FIGURE 7:**
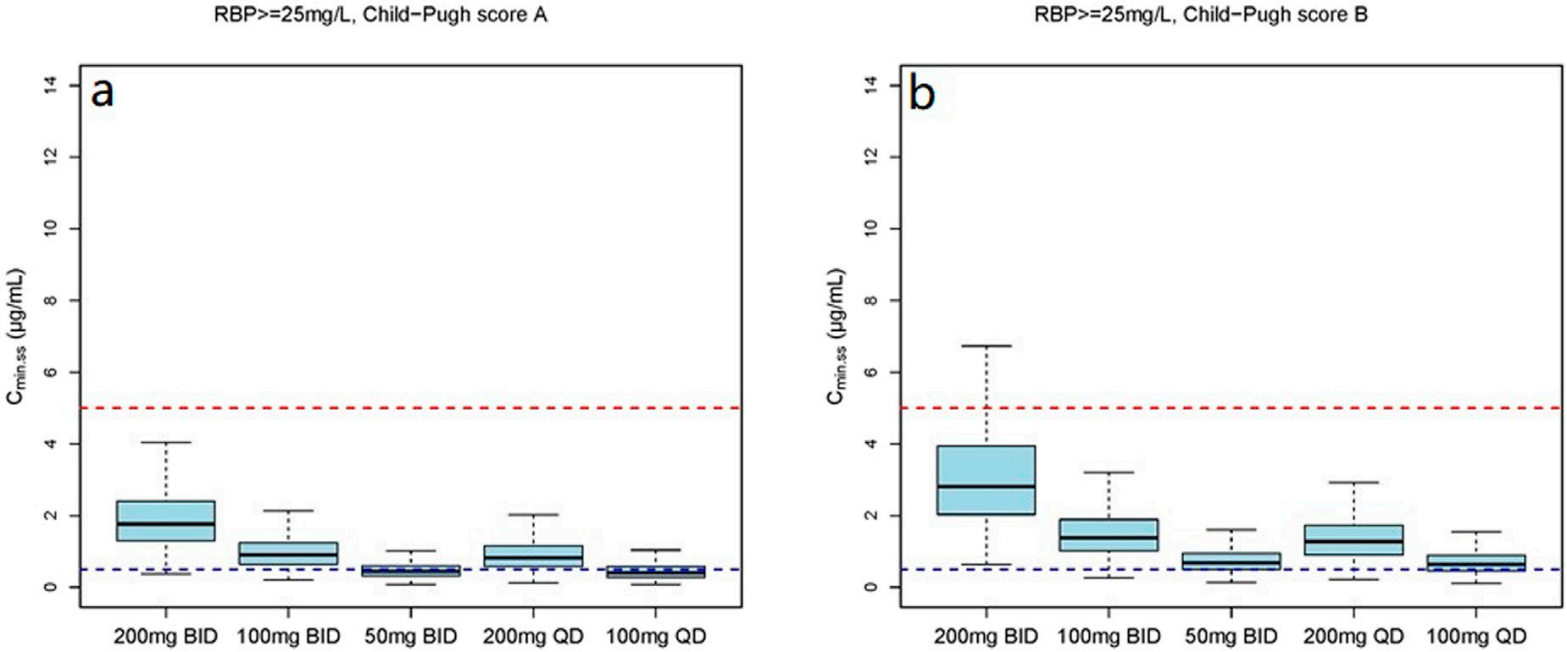
The C_min,ss_ distribution under different dosage regimens in subjects with RBP ≥25 mg/L. **(a)** The C_min,ss_ distribution in subjects with Child-Pugh score A; **(b)** The C_min,ss_ distribution in subjects with Child-Pugh score B.

The simulation results showed a negative correlation between VRC exposure and patient RBP levels (Figures 5–7). A comparative analysis of compliance rates was further conducted based on the Child-Pugh grades and RBP levels. In individuals with RBP <25 mg/L, the optimal dosage regime was 200 mg/d for those classified as Child-Pugh A, with a compliance rate of 95.5%–96.7%. In cases of Child-Pugh B, the compliance rates for the 200 mg/d and 100 mg/d regimes were above 90.0% (92.3%–93.8% vs. 93.1%–93.9%), while for the Child-Pugh C group, the most effective dosage regime was 100 mg/d, with a compliance rate of 97.6%–97.8%. At RBP levels ≥25 mg/L, the optimal dosage regimes were 200 mg/d for Child-Pugh A and 100 mg/d for Child-Pugh B groups (Supplementary Table S7).

## 4 Discussion

The high inter-individual variability and unpredictability of VRC present significant challenges for effective clinical medication management. TDM is a useful tool in precision medicine, aimed at achieving optimal therapeutic efficacy while concurrently minimizing the risk of toxicity. The PPK model provides a quantitative framework for analyzing the variables affecting PK parameters, thereby providing a useful tool for developing individualized therapeutic approaches. Our research represents the first external and systematic assessment of the predictive efficacy of the PPK model for VRC within a cirrhosis population. Notably, RBP was identified as a promising novel marker for initial dose optimization of VRC. In addition, the optimal regimens were recommended.

A one-compartment model featuring first-order absorption and first-order elimination was used to elucidate the PK characteristics of VRC. Based on data from the exploratory analysis, the results showed that none of the included covariates (age, WT, eGFR, PCTand CRP) had a significant influence on the PK of VRC, which were not consistent with previous studies (Shi C et al., 2019; Chantharit et al., 2020). In addition, the liver function parameters TBA, TBIL and DBIL had a significant impact on the CL of VRC in the forward selection while were excluded in the final model. This observation could be attributed to the qualitative nature of the indicators of liver impairment, rather than a quantitative assessment of the effect of this impairment on elimination of the drug by the liver (Dvorackova et al., 2023).

Child-Pugh classification is currently the most widely accepted grading system for investigating PK in hepatic impairment (Palmieri and Macpherson, 2019). Our findings indicate that the Child-Pugh grade showed significantly influence on the CL of VRC (ΔOFV = −38.366), which was consistent with previous findings (Ren et al., 2019; Lin et al., 2020). The CL of VRC in the cirrhosis population was 6.96 L/h, and comparable to previous studies, which reported a CL range of 6.63–7.35 (Chantharit et al., 2020; Hu et al., 2023; Chen et al., 2019). However, other studies on liver dysfunction populations have reported VRC clearance values of 0.88–3.31 L/h, which is significantly lower than our results (Ren et al., 2019; Lin et al., 2020; Tang et al., 2020). This phenomenon could be attributed to the saturated metabolism of VRC in the liver, with the hepatic function of the enrolled patients contributing to CL variations. In addition, although Child-Pugh classification serves as an indication of the severity of liver disease, this system does not express quantitative changes in hepatic metabolic function responsible for drug clearance (Schuppan and Afdhal, 2008).

Monte Carlo Simulations were conducted and patients administered dosage recommendations based on Child-Pugh grades. Both the 400 and 200 mg/d dosage regimes achieved a compliance rate of C_min,ss_> 94.0% for patients classified as Child-Pugh A. For Child-Pugh B patients, the recommended dosage was 200 mg/d, resulting in a compliance rate of C_min,ss_> 98.0%. In the case of Child-Pugh C patients, both the 200 and 100 mg/d dosage regimes were recommended, with a compliance rate of C_min,ss_> 90.0%. However, the recommended dosage regimes for Child-Pugh A and C patients were not fully consistent with the package insert and previous findings (VFEND, 2022; Yamada et al., 2018). Numerous studies have demonstrated that a dosage of 300 mg administered intravenously twice daily may be more effective in meeting therapeutic needs (Dvorackova et al., 2023; Lin et al., 2020), while a maintenance dose of 100 mg once daily of voriconazole is recommended for acute-on-chronic liver failure (ACLF) patients (Gao et al., 2018), consistent with our conclusions. However, due to the nonlinear pharmacokinetics resulting from the saturation metabolism of VRC, PPK model-guided TDM is crucial for achieving individualized precision in therapeutic management.

RBP is a low molecular weight protein which mainly synthesized in the liver, and the liver cirrhosis could lowers the liver’s ability to synthesize RBP (Domingos et al., 2016; Chaves et al., 2015). The results of our study firstly reviewed that the RBP is positively correlated with CL of VRC, with the influence coefficient of 0.428 in the final PK model (ΔOFV = −35.299). Previous studies showed that serum levels of RBP are reduced in chronic liver disease and are linked with the Child-Pugh score of disease severity (Chaves et al., 2015; Tacke et al., 2008; Tan et al., 2018), which might be one of the mechanisms by which RBP affects the CL of VRC. However, further research is needed to illustrate the exact mechanism.

The mean (SD) RBP level of enrolled patients was 27.1 (24.9) mg/L and the median concentration was 23.9 mg/L. In view of these findings, we stratified RPB levels based on approximate median values. The dosage simulation results indicated that at RBP <25 μg/mL, the recommended doses were 200 mg/d and 100 mg/d for patients in the Child-Pugh A and C groups, respectively. Furthermore, both C_min,ss_ compliance rates of 200 mg/d and 100 mg/d regimes were >92.0% for Child-Pugh B patients, while the rates of C_min,ss_<0.5 mg/L and C_min,ss_>5.0 mg/L were variable. At RBP ≥25 μg/mL, the recommended dosages were 400 mg/d and 200 mg/d for Child-Pugh A and B patients, respectively. The research initially provided a detailed dosage regime tailored to Child-Pugh grades and RBP levels of cirrhosis patients, which was not fully consistent with the package insert of VRC. RBP is primarily synthesized by the rough endoplasmic reticulum of liver cells, indicating higher specificity and sensitivity as a marker of liver function damage (Tacke et al., 2008). Numerous previous studies have demonstrated that RBP levels are significantly reduced in cirrhosis and negatively correlated with Child-Pugh scores (Tacke et al., 2008; Brissot et al., 1978; Chaves et al., 2015; Tan et al., 2018; Zhang et al., 2014), which could further support our dosage recommendations. However, the results of our clinical RBP distribution analysis (Supplementary Table S6) showed that the 90% confidence interval (CI), Mean and Median RBP level of the Child-Pugh C patients in our study were 3.1–24.1 mg/L, 14 (26.5) mg/L and 6.4 mg/L, respectively. In addition, previous investigation that the median RBP level in patients classified as Child-Pugh C is significantly lower than 25 mg/L (Zhang et al., 2014), which is consistent with our study. Additionally, the sample size of the RBP above 25 mg/L is also limited. Therefore, our study specifically focused on the C_min,ss_ compliance rates of Child-Pugh C patients with RPB levels below 25 mg/L. However, further studies are necessary to authenticate our findings.

Patient compliance is crucial for the successful management of disease outcomes. Additionally, we conducted a comparative analysis of exposure across different maintenance dosing regimens. Consistent with previous studies,our results showed no significant differences in exposure under the same daily maintenance dosing regimen (Trang et al., 2017). The terminal elimination half-life (t_1/2_) of VRC is reported as 6 h in healthy individuals, as stated in the package insert. However, previous studies have shown that t_1/2_ could extend to 53.1 h (Weiler et al., 2007) and 117.2 h (Tan et al., 2018). Thus, the once-daily dosage schedule of VRC could be given for the cirrhosis population in order to improve patient compliance.

Our study has a number of limitations that should be taken into consideration. Firstly, the influence of phenotypic factors on the metabolism of VRC, such as CYP2C19 and CYP2C9, requires further investigation. Secondly, the lack of comprehensive data on VRC-related adverse events (AEs) hinders our ability to establish a relationship between AEs and C_min,ss_. Finally, the study was based on C_min,ss_ samples alone and the sample size was limited, highlighting the necessity for a larger sample size and more robust sampling model for further validation.

## 5 Conclusion

In conclusion, our retrospective study led to the successful development of a PPK model of VRC in patients with cirrhosis based on real-world clinical data, which demonstrates good stability and accuracy. Firstly, this study represents the first external and systematic assessment of the predictive efficacy of the VRC PPK model in a cirrhosis population, yielding mean prediction error (MPE) and mean absolute prediction error (MAPE) of −0.84% and 28.89%, respectively. Our results highlight the strong predictive performance of the PPK model. Secondly, Child-Pugh classification and RBP emerged as covariates that had a significant influence on the CL of VRC and RBP was identified as a novel marker exhibiting an influence coefficient of 0.428 on VRC clearance. Thirdly, based on the model, a dosage simulation recommendation was conducted. For patients with RBP levels ≥25 mg/L, the recommended doses for Child-Pugh A and B groups were 400 mg/d and 200 mg/d, respectively. At RBP levels <25 mg/L, the recommended dosages for Child-Pugh A and C patients were 200 mg/d and 100 mg/d, respectively. In the case of Child-Pugh B patients, both 200 mg/d and 100 mg/d were recommended, which need for careful monitoring of the recommended concentration ranges. In addition, the analysis revealed no significant differences in drug exposure under the same daily maintenance dosing regimen. Thus, a once-daily VRC dosage for the cirrhosis population may improve patient compliance and enhance treatment outcomes. While many unexplained factors could contribute to variations in the PK characteristics of VRC, studies with a larger sample size, multivariate approaches, and dense sampling models are necessary for further PPK validation. Furthermore, model-guided TDM is crucial for achieving individualized precision medication.

## Data Availability

The original contributions presented in the study are included in the article/Supplementary Material, further inquiries can be directed to the corresponding authors.

## Abbreviations

AGE, age; ALT, alanine aminotransferase; ANOVA, analysis of variance; AST, aspartate aminotransferase; BMI, body mass index; BSA, body surface area; Cmin, minimum concentration; CR, creatinine; CRCL, creatinine clearance; CWRES, conditional weighted residuals; DV, dependent variable; GOF, goodness-of-fit; HGB, hemoglobin; HT, height; IIV, inter-individual variability; IOV, inter-occasion variability; MDV, missing dependent variable; OFV, objective function value; PK, pharmacokinetics; PPK, population pharmacokinetics; PRED, prediction; RUV, residual unexplained variability; SEX, sex; TBIL, total bilirubin; WT, weight.

## References

[B1] Asrani S. K. Devarbhavi H. Eaton J. Kamath P. S. (2019). Burden of liver diseases in the world. J. Hepatol. 70, 151–171. 10.1016/j.jhep.2018.09.014 30266282

[B2] Brissot P. Le Treut A. Dien G. Cottencin M. Simon M. Bourel M. (1978). Hypovitaminemia A in idiopathic hemochromatosis and hepatic cirrhosis. Role of retinol-binding protein and zinc. Digestion 17, 469–478. 10.1159/000198153 568576

[B3] Chantharit P. Tantasawat M. Kasai H. Tanigawara Y. (2020). Population pharmacokinetics of voriconazole in patients with invasive aspergillosis: serum albumin level as a novel marker for clearance and dosage optimization. Ther. Drug Monit. 42, 872–879. 10.1097/ftd.0000000000000799 32947557

[B4] Chaves G. V. Peres W. A. Gonçalves J. C. Ramalho A. (2015). Vitamin A and retinol-binding protein deficiency among chronic liver disease patients. Nutrition 31, 664–668. 10.1016/j.nut.2014.10.016 25837210

[B5] Chen C. Yang T. Li X. Ma L. Liu Y. Zhou Y. (2019). Population pharmacokinetics of voriconazole in Chinese patients with hematopoietic stem cell transplantation. Eur. J. Drug Metab. Ph. 44, 659–668. 10.1007/s13318-019-00556-w 31041728

[B6] Chen K. Zhang X. Ke X. Du G. Yang K. Zhai S. (2018). Individualized medication of voriconazole: a practice guideline of the division of therapeutic drug monitoring, Chinese Pharmacological Society. Ther. Drug Monit. 40, 663–674. 10.1097/ftd.0000000000000561 30192314 PMC6250289

[B7] Domingos M. A. Moreira S. R. Gomez L. Goulart A. Lotufo P. A. Benseñor I. etal (2016). Urinary retinol-binding protein: relationship to renal function and cardiovascular risk factors in chronic kidney disease. PLoS One 11, e0162782. 10.1371/journal.pone.0162782 27655369 PMC5031461

[B8] Dvorackova E. Sima M. Vyskocilova K. Kotowski T. Dunovská K. Klapkova E. (2023). Population pharmacokinetics and covariate-based dosing individualization of voriconazole in lung transplant recipients. J. Chemother. 36, 35–44. 10.1080/1120009x.2023.2219590 37272077

[B9] Fernández J. Piano S. Bartoletti M. Wey E. Q. (2021). Management of bacterial and fungal infections in cirrhosis: the MDRO challenge. J. Hepatol. 75, S101–S117. 10.1016/j.jhep.2020.11.010 34039482

[B10] Gao J. Zhang Q. Wu Y. Li Y. Qi T. Zhu C. (2018). Improving survival of acute-on-chronic liver failure patients complicated with invasive pulmonary aspergillosis. Sci. Rep. 8, 876–883. 10.1038/s41598-018-19320-2 29343867 PMC5772638

[B11] Gustot T. Felleiter P. Pickkers P. Sakr Y. Rello J. Velissaris D. (2014). Impact of infection on the prognosis of critically ill cirrhotic patients: results from a large worldwide study. Liver Int. 34, 1496–1503. 10.1111/liv.12520 24606193

[B12] Hu L. Huang S. Huang Q. Huang J. Feng Z. He G. (2023). Population pharmacokinetics of voriconazole and the role of CYP2C19 genotype on treatment optimization in pediatric patients. PLoS ONE 18, e0288794. 10.1371/journal.pone.0288794 37695751 PMC10495004

[B13] ICH (2022). Bioanalytical method validation and study sample analysis. Available online at: https://www.ich.org/page/multidisciplinary-guidelines.

[B14] Lin X. B. Huang F. Tong L. Xia Y. Z. Wu J. J. Li J. (2020). Pharmacokinetics of intravenous voriconazole in patients with liver dysfunction: a prospective study in the intensive care unit. Int. J. Infect. Dis. 93, 345–352. 10.1016/j.ijid.2020.02.041 32109625

[B15] Maraolo A. E. Scotto R. Zappulo E. Pinchera B. Schiano Moriello N. Nappa S. (2020). Novel strategies for the management of bacterial and fungal infections in patients with liver cirrhosis: focus on new antimicrobials. Expert Rev. anti-inf. 18, 191–202. 10.1080/14787210.2020.1725473 32011191

[B16] Palmieri C. Macpherson I. (2019). Use of the Child-Pugh score in anticancer drug dosing decision making: proceed with caution. Lancet Oncol. 20, e289. 10.1016/S1470-2045(19)30296-7 31162096

[B17] Ren Q. X. Li X. G. Mu J. S. Bi J. F. Du C. H. Wang Y. H. (2019). Population pharmacokinetics of voriconazole and optimization of dosage regimens based on Monte Carlo simulation in patients with liver cirrhosis. J. Pharm. Sci. 108, 3923–3931. 10.1016/j.xphs.2019.09.019 31562869

[B18] Schuppan D. Afdhal N. H. (2008). Liver cirrhosis. Lancet 371, 838–851. 10.1016/s0140-6736(08)60383-9 18328931 PMC2271178

[B19] Shi C. Xiao Y. Mao Y. Wu J. Lin N. (2019). Voriconazole: a review of population pharmacokinetic analyses. Clin. Pharmacokinet. 58, 687–703. 10.1007/s40262-019-00735-7 30687893

[B20] Tacke F. Weiskirchen R. Trautwein C. (2008). Liver function critically determines serum retinol-binding protein 4 (RBP4) levels in patients with chronic liver disease and cirrhosis. Hepatology 48, 1724–1725. 10.1002/hep.22544 18972556

[B21] Tan L. Meng Y. Zeng T. Wang Q. Long T. Wu S. (2018). Clinical diagnostic significance of prealbumin, cholinesterase and retinol binding protein in liver cirrhosis combined with encephalopathy. Br. J. Biomed. Sci. 76, 24–28. 10.1080/09674845.2018.1523673 30392460

[B22] Tang D. Song B. Yan M. Zou J. Zhang M. Zhou H. (2019). Identifying factors affecting the pharmacokinetics of voriconazole in patients with liver dysfunction: a population pharmacokinetic approach. Basic Clin. Pharmacol. 125, 34–43. 10.1111/bcpt.13208 30715804

[B23] Tang D. Yan M. Song B. Zhao Y. C. Xiao Y. Wang F. (2020). Population pharmacokinetics, safety and dosing optimization of voriconazole in patients with liver dysfunction: a prospective study. Br. J. Clin. Pharmacol. 87, 1890–1902. 10.22541/au.158880237.70676434 33010043

[B24] Trang M. Dudley M. N. Bhavnani S. M. (2017). Use of Monte Carlo simulation and considerations for PK-PD targets to support antibacterial dose selection. Curr. Opin. Pharmacol. 36, 107–113. 10.1016/j.coph.2017.09.009 29128853

[B25] Verma N. Singh S. Singh M. Chauhan A. Pradhan P. Jaiswal N. (2021). Global epidemiological burden of fungal infections in cirrhosis patients: a systematic review with meta‐analysis. Mycoses 65, 266–284. 10.1111/myc.13387 34724269

[B26] VFEND (Voriconazole) (2022). New York, NY: Pfizer Inc.

[B27] Wang T. Yan M. Tang D. Dong Y. Zhu L. Du Q. (2021). Using Child‐Pugh class to optimize voriconazole dosage regimens and improve safety in patients with liver cirrhosis: insights from a population pharmacokinetic model‐based analysis. Pharmacotherapy 41, 172–183. 10.1002/phar.2474 33064889

[B28] Wang T. Yan M. Tang D. Xue L. Zhang T. Dong Y. (2018a). Therapeutic drug monitoring and safety of voriconazole therapy in patients with Child-Pugh class B and C cirrhosis: a multicenter study. Int. J. Infect. Dis. 72, 49–54. 10.1016/j.ijid.2018.05.009 29793038

[B29] Wang T. Yan M. Tang D. Xue L. Zhang T. Dong Y. (2018b). A retrospective, multicenter study of voriconazole trough concentrations and safety in patients with Child-Pugh class C cirrhosis. J. Clin. Pharm. Ther. 43, 849–854. 10.1111/jcpt.12724 29893015

[B30] Weiler S. Zoller H. Graziadei I. Vogel W. Bellmann-Weiler R. Joannidis M. (2007). Altered pharmacokinetics of voriconazole in a patient with liver cirrhosis. Antimicrob. Agents Chem. 51, 3459–3460. 10.1128/AAC.00791-07 PMC2043231 17606679

[B31] Yamada T. Imai S. Koshizuka Y. Tazawa Y. Kagami K. Tomiyama N. (2018). Necessity for a significant maintenance dosage reduction of voriconazole in patients with severe liver cirrhosis (Child-Pugh class C). Biol. Pharm. Bull. 41, 1112–1118. 10.1248/bpb.b18-00164 29760306

[B32] Zhang X. Wu J. Ye B. (2014). Correlation analysis of serum retinol binding protein level and liver cirrhosis Child-Pugh hierarchy. Mod. Med. J. China 16, 17–19. 10.3969/j.issn.1672-9463.2014.08.004

