# Supplementary material for: Serum retinol binding protein as a novel marker for clearance and dosage optimization: pharmacokinetics study of voriconazole in a cirrhosis population
Source: Front Pharmacol. 2025 May 21;16:1543323. doi: 10.3389/fphar.2025.1543323 (PMC12133530; doi:10.3389/fphar.2025.1543323)
Supplement: Supplementary file 1 [file DataSheet1.doc]

**Supplementary Materials**

Table S1 Patients Characteristic of the PK model

| Characteristic | Total (N=78) | Characteristic | Total (N=78) |
| --- | --- | --- | --- |
| Classification covariate | | | |
| Sex | | Proton pump inhibitors(PPI) combination | |
| male | 57 (73.1%) | No | 30 (38.5%) |
| female | 21 (26.9%) | Yes | 48 (61.5%) |
| Tuberculosis drugs combination | | After liver transplantation | |
| No | 70 (89.7%) | No | 67 (85.9%) |
| Yes | 8 (10.3%) | Yes | 11 (14.1%) |
| Child-Pugh Classification | |  | |
| A | 30 (38.5%) |  |  |
| B | 20 (25.6%) |  |  |
| C | 28 (35.9%) |  |  |
| Continuous covariate | | | |
| Age | | ALT | |
| Mean (SD) | 54.4 (14.0) | Mean (SD) | 38.8 (39.4) |
| Median [Min, Max] | 55.5 [27.0, 91.0] | Median [Min, Max] | 23.0 [4.00, 171] |
|  |  | Missing | 1 (1.3%) |
| Height | | AST | |
| Mean (SD) | 170 (8.15) | Mean (SD) | 65.3 (82.2) |
| Median [Min, Max] | 170 [152, 185] | Median [Min, Max] | 35.0 [11.0, 494] |
| Missing | 3 (3.8%) | Missing | 1 (1.3%) |
| WT | | TBIL | |
| Mean (SD) | 64.9 (14.3) | Mean (SD) | 135 (183) |
| Median [Min, Max] | 63.0 [41.0, 120] | Median [Min, Max] | 32.0 [2.30, 634] |
| Missing | 10 (12.8%) | Missing | 1 (1.3%) |
| BMI | | DBIL | |
| Mean (SD) | 22.5 (4.37) | Mean (SD) | 101 (138) |
| Median [Min, Max] | 22.3 [15.8, 39.2] | Median [Min, Max] | 24.1 [0.900, 484] |
| Missing | 10 (12.8%) | Missing | 1 (1.3%) |
| BSA | | ALP | |
| Mean (SD) | 1.74 (0.197) | Mean (SD) | 143 (99.8) |
| Median [Min, Max] | 1.74 [1.39, 2.32] | Median [Min, Max] | 109 [43.0, 526] |
| Missing | 10 (12.8%) | Missing | 3 (3.8%) |
| CRP | | TBA | |
| Mean (SD) | 37.6 (39.4) | Mean (SD) | 65.6 (84.9) |
| Median [Min, Max] | 21.9 [0.680, 163] | Median [Min, Max] | 9.70 [1.50, 347] |
| Missing | 19 (24.4%) | Missing | 3 (3.8%) |
| WBC | | CR | |
| Mean (SD) | 7.58 (6.51) | Mean (SD) | 102 (80.7) |
| Median [Min, Max] | 5.59 [0.130, 38.6] | Median [Min, Max] | 74.0 [36.0, 460] |
| Missing | 1 (1.3%) | Missing | 1 (1.3%) |
| percentage of neutrophils (NE%) | | CRCL | |
| Mean (SD) | 70.6 (22.4) | Mean (SD) | 84.1 (35.6) |
| Median [Min, Max] | 76.8 [0, 97.7] | Median [Min, Max] | 94.1 [11.6, 135] |
| Missing | 1 (1.3%) | Missing | 2 (2.6%) |
| HGB | | RBP | |
| Mean (SD) | 92.1 (24.7) | Mean (SD) | 27.1 (24.9) |
| Median [Min, Max] | 95.0 [44.0, 159] | Median [Min, Max] | 23.9 [2.50, 137] |
| Missing | 1 (1.3%) | Missing | 7 (9.0%) |
| PCT | | eGFR | |
| Mean (SD) | 1.71 (2.18) | Mean (SD) | 96.2 (54.9) |
| Median [Min, Max] | 0.780 [0.0300, 9.27] | Median [Min, Max] | 89.5 [15.8, 269] |
| Missing | 17 (21.8%) | Missing | 11 (14.1%) |
| ALB | | GGT | |
| Mean (SD) | 32.9 (4.89) | Mean (SD) | 120 (148) |
| Median [Min, Max] | 32.3 [21.9, 43.7] | Median [Min, Max] | 59.0 [13.0, 794] |
| Missing | 1 (1.3%) | Missing | 3 (3.8%) |

Table S2 Patients Characteristic of the external validation

| Characteristic | Total (N=22) | Characteristic | Total (N=22) |
| --- | --- | --- | --- |
| Classification covariate | | | |
| Sex | | Child-Pugh Classification | |
| male | 10 (45.5%) | A | 11 (50.0%) |
| female | 12 (54.5%) | B | 6 (27.3%) |
|  |  | C | 5 (22.7%) |
| Continuous covariate | | | |
| Age | | ALT | |
| Mean (SD) | 51.1 (9.76) | Mean (SD) | 42.0 (52.5) |
| Median [Min, Max] | 51.0 [37.0, 74.0] | Median [Min, Max] | 28.0 [11.0, 249] |
| Height | | AST | |
| Mean (SD) | 165 (8.65) | Mean (SD) | 53.5 (51.1) |
| Median [Min, Max] | 163 [152, 180] | Median [Min, Max] | 38.5 [8.00, 195] |
| Missing | 1 (4.5%) |  |  |
| WT | | TBIL | |
| Mean (SD) | 63.1 (17.6) | Mean (SD) | 132 (226) |
| Median [Min, Max] | 60.0 [40.0, 92.0] | Median [Min, Max] | 24.2 [4.50, 751] |
| Missing | 1 (4.5%) |  |  |
| BMI | | DBIL | |
| Mean (SD) | 22.8 (4.85) | Mean (SD) | 93.8 (157) |
| Median [Min, Max] | 22.1 [15.8, 31.4] | Median [Min, Max] | 16.1 [1.30, 525] |
| Missing | 1 (4.5%) | Missing |  |
| BSA | | ALP | |
| Mean (SD) | 1.69 (0.254) | Mean (SD) | 142 (56.2) |
| Median [Min, Max] | 1.65 [1.33, 2.10] | Median [Min, Max] | 138 [37.0, 232] |
| Missing | 1 (4.5%) | Missing | 2 (9.1%) |
| CRP | | TBA | |
| Mean (SD) | 31.7 (22.0) | Mean (SD) | 78.1 (112) |
| Median [Min, Max] | 22.7 [14.2, 86.6] | Median [Min, Max] | 8.45 [2.30, 316] |
| Missing | 6 (27.3%) | Missing | 2 (9.1%) |
| WBC | | CR | |
| Mean (SD) | 8.10 (7.01) | Mean (SD) | 68.0 (27.3) |
| Median [Min, Max] | 5.38 [1.27, 27.7] | Median [Min, Max] | 64.0 [26.0, 149] |
| percentage of neutrophils (NE%) | | CRCL | |
| Mean (SD) | 65.1 (17.9) | Mean (SD) | 98.1 (25.3) |
| Median [Min, Max] | 68.6 [33.3, 95.9] | Median [Min, Max] | 101 [47.8, 139] |
| HGB | | RBP | |
| Mean (SD) | 97.4 (28.2) | Mean (SD) | 31.4 (23.1) |
| Median [Min, Max] | 96.5 [45.0, 154] | Median [Min, Max] | 25.0 [2.00, 78.0] |
| PCT | | eGFR | |
| Mean (SD) | 0.704 (0.737) | Mean (SD) | 106 (48.7) |
| Median [Min, Max] | 0.510 [0.0800, 2.66] | Median [Min, Max] | 102 [27.6, 237] |
| Missing | 4 (18.2%) | Missing | 1 (4.5%) |
| ALB | | GGT | |
| Mean (SD) | 34.6 (5.26) | Mean (SD) | 107 (92.3) |
| Median [Min, Max] | 34.2 [23.9, 44.0] | Median [Min, Max] | 77.0 [21.0, 389] |
|  |  | Missing | 2 (9.1%) |

Table S3 CL and V estimations with different Ka values

|  | **Original** | **0.5 times** | **0.75 times** | **1.25 times** | **2 times** | **Non-fix** |
| --- | --- | --- | --- | --- | --- | --- |
| **K_a_ values (h^-1^)** | **1.1 (Fix)** | **0.55 (Fix)** | **0.825 (Fix)** | **1.375 (Fix)** | **2.2 (Fix)** | **1.1E+07** |
| CL estimates (L/h) | 6.96 | 7 | 6.97 | 6.96 | 6.95 | 6.93 |
| Changes (%) | / | 0.57 | 0.14 | 0 | 0.14 | 0.43 |
| V estimates (L) | 745 | 749 | 746 | 745 | 745 | 743 |
| Changes (%) | / | 0.54 | 0.13 | 0 | 0 | 0.27 |

*CL*: clearance; *V*: volume of distribution

Table S4 The process table of establishing PPK model for VRC

| **No.** | **file name** | **Model description** | **objective function value** | **Reference model** | **Change value of objective function** | **Significant** |
| --- | --- | --- | --- | --- | --- | --- |
| 1 | model01 | 1 compartment model with linear elimination | 462.047 | - | - | - |
| 2 | model02 | model01-IIVVC | 462.048 | model01 | 0.001 | NO |
| 3 | model03 | 2 compartment model with linear elimination | 462.046 | model01 | -0.001 | NO |
| 4 | model04 | model01 + F | 459.362 | model01 | -2.685 | NO |
| 5 | model05 | model04-IIVVC | 459.362 | model04 | 0 | NO |
| 6 | model06 | model01+D1 | 462.74 | model01 | 0.693 | NO |
| 7 | model07 | model02 + CPUGH | 423.682 | model01 | -38.366 | YES |
| 8 | model08 | model07 + CLBMI | 422.333 | model07 | -1.349 | NO |
| 9 | model09 | model07 + CLBSA | 422.234 | model07 | -1.488 | NO |
| 10 | model10 | model07 + CLCRCL | 422.518 | model07 | -1.164 | NO |
| 11 | model11 | model07 + CLAGE | 419.609 | model07 | -4.073 | NO |
| 12 | model12 | model07 + CLHT | 422.272 | model07 | -1.41 | NO |
| 13 | model13 | model07 + CLWT | 422.025 | model07 | -1.657 | NO |
| 14 | model14 | model07 + CLCRP | 423.681 | model07 | -0.001 | NO |
| 15 | model15 | model07 + CLWBC | 422.835 | model07 | -0.847 | NO |
| 16 | model16 | model07 + CLHb | 420.411 | model07 | -3.271 | NO |
| 17 | model17 | model07 + CLPCT | 422.403 | model07 | -1.279 | NO |
| 18 | model18 | model07 + CLALB | 423.576 | model07 | -0.106 | NO |
| 19 | model19 | model07 + CLALT | 423.476 | model07 | -0.206 | NO |
| 20 | Model20 | model07 + CLAST | 417.674 | model07 | -6.008 | NO |
| 21 | model21 | model07 + CLTBIL | 417.747 | model07 | -8.935 | YES |
| 22 | model22 | model07 + CLDBIL | 412.436 | model07 | -11.246 | YES |
| 23 | model23 | model07 + CLGGT | 421.126 | model07 | -2.556 | NO |
| 24 | model24 | model07 + CLALP | 423.393 | model07 | -0.289 | NO |
| 25 | model25 | model07 + CLTBA | 395.442 | model07 | -28.24 | YES |
| 26 | model26 | model07 + CLCREAT | 423.556 | model07 | -0.116 | NO |
| 27 | model27 | model07 + CLGFR | 423.154 | model07 | -0.528 | NO |
| 28 | model28 | Final model: model07 + CLRBP | 388.383 | model07 | -35.299 | YES |
| 29 | model29 | model07 + CLPPI | 423.377 | model07 | 0.305 | NO |
| 30 | model30 | model07 + CLJH | 422.289 | model07 | -1.393 | NO |
| 31 | model31 | model07 + CLGYZ | 420.008 | model07 | -3.647 | NO |
| 32 | model32 | model07 + CLGYZ | 412.180 | model07 | -2.502 | NO |
| 33 | model33 | model28 + CLTBIL | 387.902 | model28 | -0.481 | NO |
| 34 | model34 | model28 + CLDBIL | 387.196 | model28 | -1.187 | NO |
| 35 | model35 | model28 + CLTBA | 382.688 | model28 | -5.695 | NO |
| 36 | model36 | model28 - CLRBP | 423.682 | model28 | 35.299 | YES |
| 37 | model37 | model28 - CPUGH | 404.72 | model28 | 16.377 | YES |

Table S5 The C_min, ss_ compliance rate of different Child-Pugh grades under different dosage regime

| **Child-Pugh grades** | **Medication interval** | **Maintain dosage** | **C _min, ss_** | | |
| --- | --- | --- | --- | --- | --- |
|  |  |  | **<0.5 mg/L** | **0.5–5.0 mg/L** | **>5.0 mg/L** |
| A | BID | 200 mg | 0% | 95.9% | 4.1% |
|  | BID | 100 mg | 5.2% | 94.8% | 0% |
|  | BID | 50 mg | 38.6% | 61.4% | 0% |
|  | QD | 200 mg | 5.6% | 94.3% | 0.1% |
|  | QD | 100 mg | 42.2% | 57.8% | 0% |
| B | BID | 200 mg | 0% | 79.9% | 20.1% |
|  | BID | 100 mg | 0.3% | 99% | 0.7% |
|  | BID | 50 mg | 10.5% | 89.5% | 0% |
|  | QD | 200 mg | 1.2% | 98.1% | 0.7% |
|  | QD | 100 mg | 13.1% | 86.9% | 0.1% |
| C | BID | 200 mg | 0% | 68.5% | 31.5% |
|  | BID | 100 mg | 0.1% | 98.3% | 1.6% |
|  | BID | 50 mg | 5% | 94.9% | 0.1% |
|  | QD | 200 mg | 0.3% | 98.2% | 1.5% |
|  | QD | 100 mg | 9.5% | 90.5% | 0% |

Table S6 The distribution of patients’ RBP level based on Child-Pugh classification

| Child-Pugh classification | A (n=30) | B (n=20) | C (n=28) | Overall (n=78) |
| --- | --- | --- | --- | --- |
| RBP (90% CI) | 7.17~63.12 | 6.93~59.82 | 3.1~24.1 | 3.48~59.07 |
| Mean (SD) | 15.7 (21.1) | 32.1 (21.1) | 14 (26.5) | 27.1 (24.9) |
| Median | 31.8 | 26.4 | 6.4 | 23.9 |

Table S7 The C_min, ss_ compliance rate of different Child-Pugh grades and RBP under different dosage regime

| **RBP level** | **Child-Pugh grades** | **Medication interval** | **Maintain dosage** | **Cmin, ss** | | |
| --- | --- | --- | --- | --- | --- | --- |
|  |  |  |  | **<0.5 mg/L** | **0.5–5.0 mg/L** | **>5.0 mg/L** |
| RBP < 25mg/L | A | BID | 200 mg | 0% | 82.3% | 17.7% |
|  |  | BID | 100 mg | 1% | 96.7% | 2.3% |
|  |  | BID | 50 mg | 18.7% | 81.2% | 0.1% |
|  |  | QD | 200 mg | 2% | 95.5% | 2.5% |
|  |  | QD | 100 mg | 22.8% | 77% | 0.2% |
|  | B | BID | 200 mg | 0% | 56.2% | 43.8% |
|  |  | BID | 100 mg | 0.1% | 92.3% | 7.6% |
|  |  | BID | 50 mg | 6.6% | 93.1% | 0.3% |
|  |  | QD | 200 mg | 0.2% | 93.8% | 6% |
|  |  | QD | 100 mg | 6% | 93.9% | 0.1% |
|  | C | BID | 200 mg | 0% | 24.3% | 75.7% |
|  |  | BID | 100 mg | 0% | 75.6% | 24.4% |
|  |  | BID | 50 mg | 0.8% | 97.6% | 1.6% |
|  |  | QD | 200 mg | 0% | 74.1% | 25.9% |
|  |  | QD | 100 mg | 1.1% | 97.8% | 1.1% |
| RBP ≥ 25mg/L | A | BID | 200 mg | 0.3% | 97.8% | 1.9% |
|  |  | BID | 100 mg | 11% | 89% | 0% |
|  |  | BID | 50 mg | 60.2% | 39.8% | 0% |
|  |  | QD | 200 mg | 18.1% | 81.9% | 0% |
|  |  | QD | 100 mg | 65.2% | 34.8% | 0% |
|  | B | BID | 200 mg | 0% | 87.6% | 12.4% |
|  |  | BID | 100 mg | 1.8% | 98% | 0.2% |
|  |  | BID | 50 mg | 25.8% | 74.2% | 0% |
|  |  | QD | 200 mg | 3.5% | 96.5% | 0% |
|  |  | QD | 100 mg | 31.9% | 68.1% | 0% |
